# Impaired renal autoregulation and pressure-natriuresis: any role in the development of heart failure in normotensive and angiotensin II-dependent hypertensive rats?

**DOI:** 10.1038/s41440-023-01401-z

**Published:** 2023-08-17

**Authors:** Zuzana Honetschlägerová, Janusz Sadowski, Elzbieta Kompanowska-Jezierska, Hana Maxová, Miloš Táborský, Petr Kujal, Luděk Červenka

**Affiliations:** 1 Center for Experimental Medicine, Institute for Clinical and Experimental Medicine, Prague, Czech Republic; 2 Department of Renal and Body Fluid Physiology, Mossakowski Medical Research Institute, Polish Academy of Sciences, Warsaw, Poland; 3 Department of Pathophysiology, 2nd Faculty of Medicine, Charles University, Prague, Czech Republic; 4 Department of Internal Medicine I, Cardiology, University Hospital Olomouc and Palacký University, Olomouc, Czech Republic; 5 Department of Pathology, 3rd Faculty of Medicine, Charles University, Prague, Czech Republic

**Keywords:** Volume-overload heart failure, Ren-2 transgenic hypertensive rat, Renal autoregulation, Renal blood flow, Sodium excretion

## Abstract

The aim of the present study was to assess the autoregulatory capacity of renal blood flow (RBF) and of the pressure-natriuresis characteristics in the early phase of heart failure (HF) in rats, normotensive and with angiotensin II (ANG II)-dependent hypertension. Ren-2 transgenic rats (TGR) were employed as a model of ANG II-dependent hypertension. HF was induced by creating the aorto-caval fistula (ACF). One week after ACF creation or sham-operation, the animals were prepared for studies evaluating in vivo RBF autoregulatory capacity and the pressure-natriuresis characteristics after stepwise changes in renal arterial pressure (RAP) induced by aortic clamping. In ACF TGR the basal mean arterial pressure, RBF, urine flow (UF), and absolute sodium excretion (U_Na_V) were all significantly lower tha n in sham-operated TGR. In the latter, reductions in renal arterial pressure (RAP) significantly decreased RBF whereas in ACF TGR they did not change. Stepwise reductions in RAP resulted in marked decreases in UF and U_Na_V in sham-operated as well as in ACF TGR, however, these decreases were significantly greater in the former. Our data show that compared with sham-operated TGR, ACF TGR displayed well-maintained RBF autoregulatory capacity and improved slope of the pressure-natriuresis relationship. Thus, even though in the very early HF stage renal dysfunction was demonstrable, in the HF model of ANG II-dependent hypertensive rat such dysfunction and the subsequent HF decompensation cannot be simply ascribed to impaired renal autoregulation and pressure-natriuresis relationship.

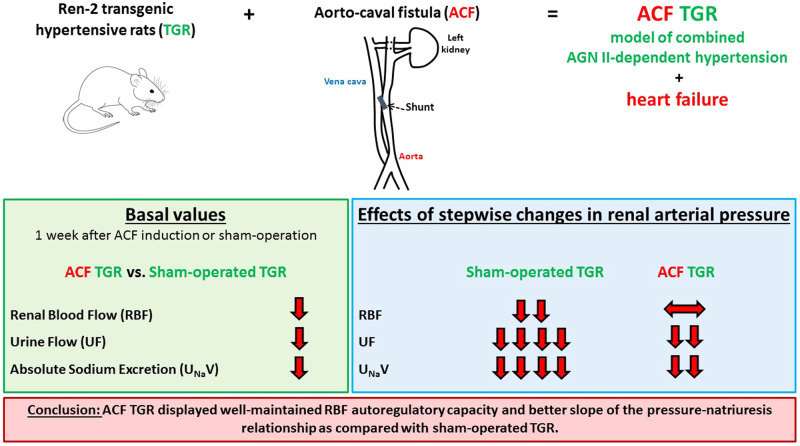

## Introduction

Heart failure (HF) is a global pandemic, affecting almost 30 million people worldwide, most frequently in developed countries; the yearly increase in the number of new patients is estimated at 1.1 million [1]. Paradoxically, the increase in the prevalence of HF is attributed, at least in part, to improved treatment of acute coronary syndromes and non-ischemic cardiovascular diseases. Remarkably, the progress in the treatment obtained with early coronary reperfusion by primary percutaneous intervention has decreased the mortality rate but not the morbidity. Somewhat surprisingly, the number of surviving patients who ultimately develop HF has augmented [2]. HF is a clinical syndrome showing progressive aggravation. The prognosis of the patients remains poor, particularly when HF is accompanied by kidney dysfunction (“cardiorenal syndrome”) [3–7], and it is generally accepted that, particularly in patients with this syndrome, there is an urgent need for new treatment strategies. This requires better understanding of the pathophysiological mechanism(s) underlying the progression of HF.

A common finding in the very early stage of HF, when cardiac functions are but minimally impaired, is a reduction of the renal blood flow (RBF) [3, 4, 8–10]. The long-held view on the role of the kidney in the progression of HF is that it is a “victim” of chronic hypoperfusion and/or of maladaptive responses to compensatory neurohormonal activation [4, 11–13]. It is agreed that the development of renal dysfunction and signs and symptoms of volume overload are hallmark features of decompensation of HF [3, 5]. While kidney condition is recognized as a prognostic marker of HF, it is thought that the therapy of HF patients should not be primarily focused on improvement of renal function [3, 5]. However, the view that the kidney is simply a victim of HF has not withstood the test of time. It does not explain many pathophysiological features of HF or the beneficial actions of new therapies for HF, such as sodium-glucose cotransporter type 2 (SGLT2) inhibition [14]. It is reminded that many clinical and experimental findings from studies performed over the past 60 years have challenged the view that the kidney is merely a victim in HF. First, Barger et al. [15] reported that abnormalities in renal sodium handling are detectable already in mild heart disease, long before overt HF is present. Second, studies performed by Brenner´s and Frohlich´s groups showed that alterations of the renal hemodynamics observed early after myocardial infarction (MI) precede the development of HF and are critically dependent on angiotensin II (ANG II) actions [16–19]. Third, Frohlich and co-workers reported that the ANG II-dependent alterations are present not only in low-output HF, i.e. HF induced by MI, but also in high-output HF, induced by chronic volume overload due to the creation of the aorto-caval fistula (ACF) [19, 20]. Unfortunately, the evidence that impairment of renal function precedes the development of HF or at least importantly contributes to its pathophysiology already in the “compensation phase” were disregarded. Recent studies in our laboratory showed that RBF is very early reduced in the hypertensive Ren-2 transgenic rat (TGR) with ACF. This is a model of severe high-output HF characterized by two major risk factors for the development of cardiorenal syndrome: hypertension and increased activity of the renin-angiotensin system (RAS) [21–23].

Considering the above evidence, we hypothesize that the kidney is not simply a victim but also a culprit of the onset decompensation and progression of HF. Specifically, we hypothesize that the impairment of autoregulation of RBF and of the pressure-natriuresis relationship is present in the very early HF phase, soon after the initial insult, and precedes the onset of HF decompensation.

Accordingly, the aim of the present study was to assess autoregulation of the RBF and the pressure-natriuresis relationships in the ACF TGR and to compare the results with those in transgene-negative, normotensive Hannover Sprague-Dawley (HanSD) rats in the early phase after creation of ACF.

## Methods

### Ethical approval and animals

The study was performed in accordance with the guidelines and practices established by the Animal Care and Use Committee of the Institute for Clinical and Experimental Medicine (IKEM), Prague, which accord with the European Convention on Animal Protection and Guidelines on Research Animal Use, and were approved by this committee and subsequently by the Ministry of Health of the Czech Republic (the decision number for this project is 18680/2020-4/OVZ). All the animals employed in the study were bred at the IKEM, which is accredited by the Czech Association for Accreditation of Laboratory Animals. Experiments were performed in heterozygous TGR that were generated by breeding male homozygous TGR with female homozygous HanSD rats. Male HanSD served as normotensive controls. Animals were housed under standard conditions (12:12, light:dark cycle) and had free access to standard rat chow and water. The study was carried out in compliance with the ARRIVE (Animals in Research: Reporting In vivo Experiments) guidelines [24].

### Heart failure model, exclusion criteria, and definition of the phase of HF

Eight-weeks-old male TGR and HanSD rats were anesthetized with intraperitoneal ketamine/midazolam mixture (Calypsol, Gedeon Richter, Hungary, 160 mg/kg and Dormicum, Roche, France, 160 mg/kg). HF variant dependent on the volume overload was then induced by creating ACF using a needle technique. This procedure is routinely performed in our laboratory and the details were reported previously [21–23, 25, 26]. Sham-operated rats underwent an identical procedure but without creating ACF (sham-ACF). If a technical error occurred during ACF creation procedure or pulsatile flow in the inferior vena cava could not be confirmed, suggesting flawed ACF function, animals were excluded from the study. An additional exclusion criterion was established based on the studies showing that after creation of ACF some rats developed major sodium retention and died within first 3–5 days after ACF creation [27, 28]. Therefore, the rats that died within the first 5 days after ACF creation were also excluded from our analyses. One week after creation of ACF, HanSD rats as well as TGR were considered to be in the phase of very early compensation phase of HF, because based on our previous studies, the transition from the compensation to the decompensation phase occurs twenty weeks after ACF creation in HanSD rats and after three weeks in TGR [21–23, 25, 29, 30].

### Renal function studies

#### Surgical preparation

On the day of experiment, rats were anesthetized with intraperitoneal sodium thiopental (50 mg/kg, i.p.) and placed on the thermoregulated surgical table to maintain body temperature at 37 °C. Tracheostomy was performed and a PE-240 tube was inserted to maintain a patent airway, and the exterior end of tracheal cannula was placed inside a small plastic chamber into which a humidified 95% oxygen/5% carbon dioxide mixture was continuously delivered. This has been shown to improve the stability of arterial blood pressure (BP) of barbiturate-anesthetized rats [31]. Notably, even if barbiturate anesthesia exhibits some negative effects on BP, we have previously reported that the values obtained in anesthetized rats are an accurate reflection of BP values in conscious rats [32–35]. The right jugular vein was cannulated with a PE-50 catheter for fluid infusion and for administration of the anesthetic. PE-50 cannulas were placed in the left carotid artery and the left femoral artery for continuous measurement of arterial BP above and below the left renal artery. Mean arterial pressure (MAP) was monitored with a pressure transducer (model MLT1050, ADInstruments) and recorded using a computerized data-acquisition system (Power Laboratory/4SP, ADInstruments). The left kidney was exposed via a flank incision, isolated from the surrounding tissue and placed in a lucite cup. The ureter was then cannulated with a PE-10 catheter. Two aortic clamps were placed on the aorta, one above the superior mesenteric artery and one below the left renal artery, to allow manipulation of renal arterial pressure (RAP). The ultrasonic transient-time flow probe (1RB, Transonic System) connected to a Transonic flowmeter was placed around the left renal artery and RBF was recorded using a computerized data-acquisition system. At the end of experiment, zero value was established by complete occlusion of the aorta. During and after the surgery, an isotonic saline solution containing bovine serum albumin (6%, Sigma Aldrich Chemical Co., Prague, Czech Republic) was infused at 60 µl/min. This general surgical preparation is based on the original methods developed by Roman and Cowley for studying pressure-natriuresis in the rat [36] and modified by Wang et al. [37]. The procedure has been standardly used in our laboratory in various hypertension models [38–42].

After completion of the surgical procedure, 50-min equilibration was allowed for rats to reach steady state before initiating one 30-min control urine collection at a baseline level of RAP. Subsequently, using the aortic clamps, RAP was increased or reduced to the levels as indicated for different experimental protocols (see below). Moreover, corresponding urine collection periods were done.

Urine volume was measured gravimetrically. The urinary sodium concentration was determined by flame photometry. The values were calculated per gram kidney weight. At the end of the experiment, whole heart weight (HW) and then left ventricle weight (LVW) (including septum), right ventricle weight (RVW) and lung weight (“wet lung weight”) were assessed as described in our previous studies [21–23, 26, 29, 30].

#### Experimental protocols

- *Control protocol in sham-operated and ACF HanSD rats, and in sham-operated and ACF TGR*.
  In all groups in which control protocol was applied, after initial control urine collection at the basal level of RAP, four additional 30-min urine collections at the same RAP were performed.
- *Experimental protocol in sham-operated HanSD rats*.
  After initial control urine collection at the basal level of RAP, four 30-min urine collections were done at RAP reduced to 110, 100, 90 and 80 mmHg. A five-min equilibration period was allowed after each RAP reduction.
- *Experimental protocol in ACF HanSD rats*.
  After initial control urine collection at the basal level of RAP, two 30-min urine collections at RAP reduced to 90 and 80 mmHg, and two 30-min urine collections at RAP increased to 110 and 120 mmHg were performed.
- Experimental protocol in sham-operated TGR.
  After initial control urine collection at the basal level of RAP, four 30-min urine collections were performed at RAP reduced to 125, 100, 90 and 80 mmHg.
- Experimental protocol in ACF TGR.

After initial control urine collection at the basal level of RAP, three 30-min urine collections were performed at RAP reduced to 100, 90 and 80 mmHg, followed by one 30-min urine collection at RAP increased to 125 mmHg.

The values of RAP steps used were chosen based on our previous studies employing sham-operated and ACF HanSD rats as well as TGR [22, 23, 29]. This was done to obtain RBF, urine flow and renal sodium excretion values as far as possible comparable between the groups of the same RAP.

The experimental protocols in all experimental groups are outlined in Table 1.

**Table 1:** Experimental groups and the values of renal arterial pressure (RAP) during individual urine collection periods (U)

*HanSD* transgene-negative Hannover Sprague-Dawley rats, *TGR* Ren-2 renin transgenic rats, *ACF* aorto-caval fistula

indicates reduction of renal arterial pressure in the appropriate experimental group and in the given urine collection period

indicates increase of renal arterial pressure in the appropriate experimental group and in the given urine collection period

### Experimental groups

The following experimental groups were examined:

1. Sham-operated HanSD rats (no ACF) + control protocol (*n* = 11)
2. Sham-operated HanSD rats + experimental protocol (*n* = 14)
3. Sham-operated TGR + control protocol (*n* = 11)
4. Sham-operated TGR + experimental protocol (*n* = 15)
5. ACF HanSD rats in very early phase of HF + control protocol (*n* = 12)
6. ACF HanSD rats in very early phase of HF + experimental protocol (*n* = 14)
7. ACF TGR in very early phase of HF + control protocol (*n* = 12)
8. ACF TGR in very early phase of HF + experimental protocol (*n* = 15)

### Histological evaluation of the heart and kidney tissues

In separate appropriately matched four experimental groups (*n* = 8 in each), i.e. sham-operated HanSD rats, sham-operated TGR, ACF HanSD rats and ACF TGR, the hearts and kidneys were subjected to histological examination of the myocardium and renal cortex as described previously [26, 43–45].

#### Heart evaluation

The rats were anesthetized with a combination of midazolam 5 mg.kg^−1^ (Dormicum, Roche Ltd., Prague, Czech Republic) and ketamine 50 mg.kg^−1^ (Calypsol, Gedeon Richter Ltd., Budapest, Hungry) i.p. The beating (pulsating) organ i.e. the native heart was perfused in situ with 20 ml of Thomas cardioplegia solution and subsequently fixed in 4% paraformaldehyde in phosphate-buffered saline and embedded into Tissue-Tek. The blocks were cut using a cryo-microtome, and cardiomyocyte width was measured in the subendocardium, mid-myocardium, and subepicardium of the LV. Cardiomyocyte length was measured only in the midmyocardium; in each layer, 30 cardiomyocytes were assessed. To avoid underestimation, only the cells in which the nucleus was visible were measured. Since there were no significant differences in the cardiomyocyte width between the layers, the data from the subendocardium, midmyocardium, and subepicardium were pooled, as was also practiced by other investigators [46]. Analysis of LV and RV fibrosis was performed in sections stained with Picrosirius red (Direct Red 80, Sigma Aldrich, MO, USA) as described in detail previously [43]. The interstitial collagen was analyzed in polarized light using 10 images of the LV and RV scanned from a midmyocardium, without perivascular areas (magnification 200x, microscope Nikon eclipse Ni-E, camera Nikon DS-L3, Tokyo, Japan). The percent area of myocardial fibrosis was calculated semiquantitatively, using imaging software NIS-Elements Ar (LIM, Prague, Czech Republic). Measurements of cardiomyocyte width and length, and of the degree of myocardial fibrosis were performed one week after either sham-operation or ACF creation. The histological examination had to be performed in separate groups of animals because perfusion with cardioplegia solution with subsequent immediate fixation in paraformaldehyde solution precludes precise determination of the whole HW and the LV or RV weights.

#### Kidney evaluation

The kidneys were used to assess glomerular damage and tubulointerstitial injury. The kidneys were fixed in 4% formaldehyde, dehydrated and embedded in paraffin. The sections stained with periodic acid, for Schiff reaction, were examined and evaluated in a blind-test fashion. Fifty glomeruli in each kidney were examined on a semi-quantitative scale. The evaluation was as follows: *grade 0*, all glomeruli normal; *grade 1*, sclerotic area up to 25% (minimal sclerosis); *grade 2*, sclerotic area 25 to 50% (moderate sclerosis); *grade 3*, sclerotic area 50 to 75% (moderate-to-severe sclerosis); *grade 4*, sclerotic area 75 to 100% (severe sclerosis). The glomerulosclerosis index (GSI) was calculated using the following formula: GSI = [(1 x n_1_) + (2 x n_2_) + (3 x n_3_) + (4 x n_4_)]/(n_0_ + n_1_ + n_2_ + n_3_ + n_4_), where n_x_ is the number of glomeruli in each grade of glomerulosclerosis. Kidney cortical tubulointerstitial injury was evaluated as defined by Nakano et al. [47], to determine inflammatory cell infiltration, tubular dilatation, atrophy, or interstitial fibrosis. The injury was graded semi-quantitatively using the following scale of lesions: grade 0, no abnormal findings; 1, mild (<25% of the cortex); 2, moderate (25–50% of the cortex); 3, severe (>50% of the cortex). The lesions were assessed in at least 30 random and non-overlapping fields in the renal cortex. Thus, the maximum score for GSI is 4 and for the index of kidney tubulointerstitial injury (TSI) is 3. The values of GSI < 0.5 and TSI < 0.4 are considered as healthy renal tissue without sign of significant renal damage. This method is always employed in our studies evaluating the degree of kidney damage [26, 44, 45].

After implementation of the exclusion criteria a total number of 156 rats (75 HanSD rats and 81 TGR) were used in all series of experiments, which means that 8 HanSD rats and 12 TGR were excluded based on the aforementioned criteria.

### Statistical analysis

All values are expressed as means ± SEM. Using the Graph-Pad Prism software (Graph Pad Software, San Diego, CA, USA), statistical analysis was performed: Student´s *t*-test for unpaired data, or one-way analysis of variance (ANOVA) followed by Tukey-Kramer multiple comparison test when appropriate. ANOVA for repeated measurements was performed for the analysis within groups (e.g. for the analysis of autoregulatory capacity of RBF). Values exceeding 95% probability limits (*p* < 0.05, two-sided) were considered statistically significant.

## Results

Table 2 summarizes the data on the basal MAP, RBF, renal vascular resistance (RVR), urine flow and absolute sodium excretion (data from periods obtained at a basal level of RAP and pooled from groups subjected to control and experimental protocols). In addition, body weights (BW) and the ratio of organ weights to BW in all four experimental groups are shown (again, pooled as stated above). Sham-operated TGR were markedly hypertensive as compared with sham-operated HanSD rats, and basal RBF, urine flow and absolute sodium excretion were also significantly higher in the former. ACF creation resulted in significant decreases in MAP in TGR as well as HanSD rats, but the BP remained significantly higher in ACF TGR, even though when expressed as percentage, decreased MAP in ACF TGR was about 37% below the level in sham-operated TGR, whereas in ACF HanSD rats the MAP was about 22% lower than in sham-operated HanSD rats (see Table 2). One week after ACF creation HanSD rats displayed marked bilateral cardiac hypertrophy, but as indicated by the ratio of RVW to LVW, the right ventricle (RV) hypertrophy was more pronounced than that of the left ventricle (LV). ACF creation in TGR caused a significant increase in the whole HW to BW ratio, but this increase was exclusively due to RV hypertrophy, because the LV to BW ratio did not increase as compared with sham-operated TGR. This explanation is further supported by a marked increase in the ratio of RVW to LVW in ACF TGR as compared with sham-operated TGR. In addition, ACF rats displayed marked lung congestion as seen from increases of wet lung weights normalized to BW, when compared with the corresponding data from their sham-operated counterparts. Notably, lung congestion was more pronounced in ACF TGR than in ACF HanSD rats (see Table 2).

**Table 2:** Basal values of arterial blood pressure, renal function and organ weights pooled from groups undergoing control and experimental protocols

	Group			
	Sham-operated HanSD	ACF HanSD	Sham-operated TGR	ACF TGR
	(*n* = 25)	(*n* = 26)	(*n* = 26)	(*n* = 27)
Body weight (g)	442 ± 8	430 ± 6	425 ± 7	431 ± 6
Mean arterial pressure (mmHg)	107 ± 2	88 ± 2^a^	156 ± 3^b^	98 ± 2^a^
Renal blood flow (ml.min^−1^.g^−1^)	8.48 ± 0.42	6.55 ± 0.22^a^	11.45 ± 0.56^b^	8.13 ± 0.29^a,c^
Renal vascular resistance (mmHg.ml^−1^.min^−1^.g^−1^)	12.62 ± 0.55	13.42 ± 0.71	13.82 ± 0.69	12.14 ± 0.64
Urine flow (µl.min^−1^.g^−1^)	18.59 ± 2.09	8.91 ± 1.19^a^	38.30 ± 3.07^b^	8.86 ± 0.98^a^
Absolute sodium excretion (µmol. min^−1^.g^−1^)	3.39 ± 0.54	0.41 ± 0.09^a^	4.77 ± 0.54^b^	0.40 ± 0.24^a^
Heart weight (mg)/Body weight (g)	2.97 ± 0.04	3.86 ± 0.11^a^	3.68 ± 0.04^b^	4.42 ± 0.07^a,c^
Left ventricle weight (mg)/Body weight (g)	2.11 ± 0.02	2.41 ± 0.02^a^	2.85 ± 0.03^b^	2.92 ± 0.04^a,c^
Right ventricle weight (mg)/Body weight (g)	0.55 ± 0.01	0.79 ± 0.02^a^	0.50 ± 0.01	0.89 ± 0.02^a,c^
Right ventricle weight (mg)/Left ventricle weight (mg)	0.259 ± 0.005	0.331 ± 0.008^a^	0.175 ± 0.003^b^	0.303 ± 0.007^a^
Lung weight (mg)/Body weight (g)	4.09 ± 0.13	5.51 ± 0.14^a^	4.12 ± 0.11	6.15 ± 0.18^a,c^

The values are the means ± SEM

*HanSD* transgene-negative Hannover Sprague-Dawley rats, *TGR* Ren-2 renin transgenic rats, *ACF* aorto-caval fistula

^a^*p* < 0.05 ACF rats vs. sham-operated rats at the same strain

^b^*p* < 0.05 Sham-operated TGR vs. sham-operated HanSD rats

^**c**^*p* < 0.05 ACF TGR vs. ACF HanSD rats

**Fig. 1:**
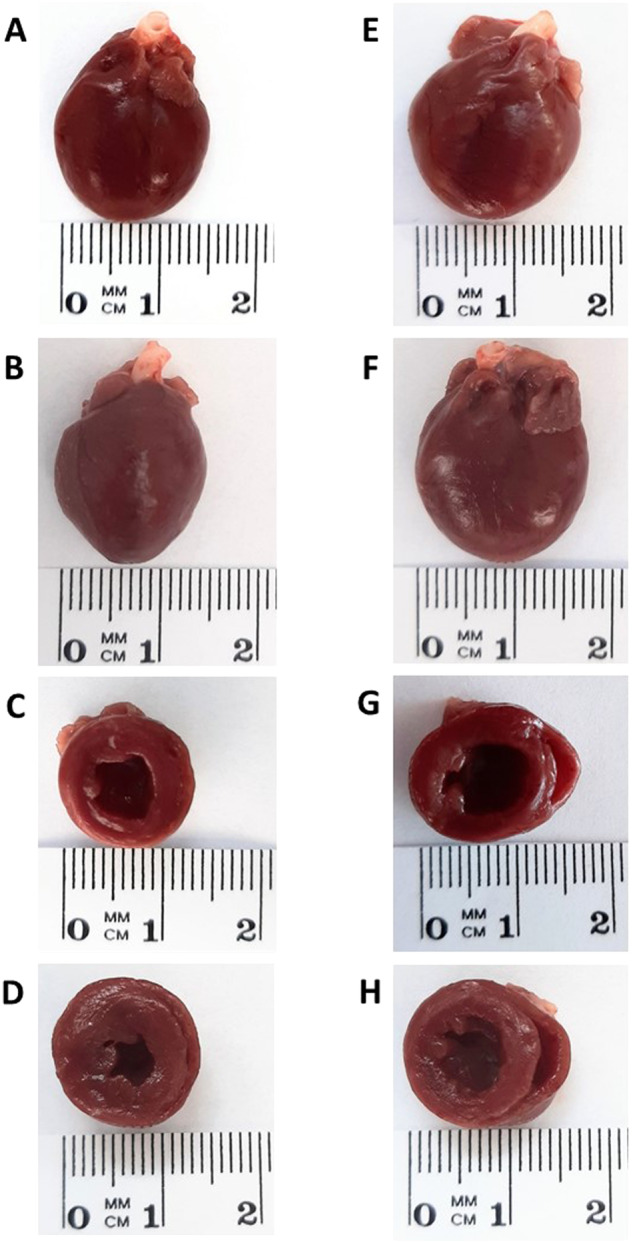
Representative images of the whole heart and transversally cut heart in sham-operated Hannover Sprague-Dawley (HanSD) rats (**A** and **C**) (average whole heart weight in this group is 1373 ± 24 mg), sham-operated Ren-2 transgenic rats (TGR) (**B** and **D**) (average whole heart weight in this group is 1561 ± 22 mg), HanSD rats with aorto-caval fistula (ACF) (**E** and **G**) (average whole heart weight in this group is 1692 ± 49 mg) and ACF TGR (**F** and **H**) (average whole heart weight in this group is 1908 ± 41 mg). All the images were obtained, one week after sham-operation or ACF creation

Gross morphological changes in the heart are shown in Fig. 1 which shows representative whole heart images in sham-operated HanSD rats, sham-operated TGR, ACF HanSD rats and ACF TGR, as well as the images of the same groups in transversely cut hearts. Sham-operated TGR displayed concentric LV hypertrophy and ACF creation caused dilatation of the LV and RV cavities accompanied by an increase of wall mass.

**Fig. 2:**
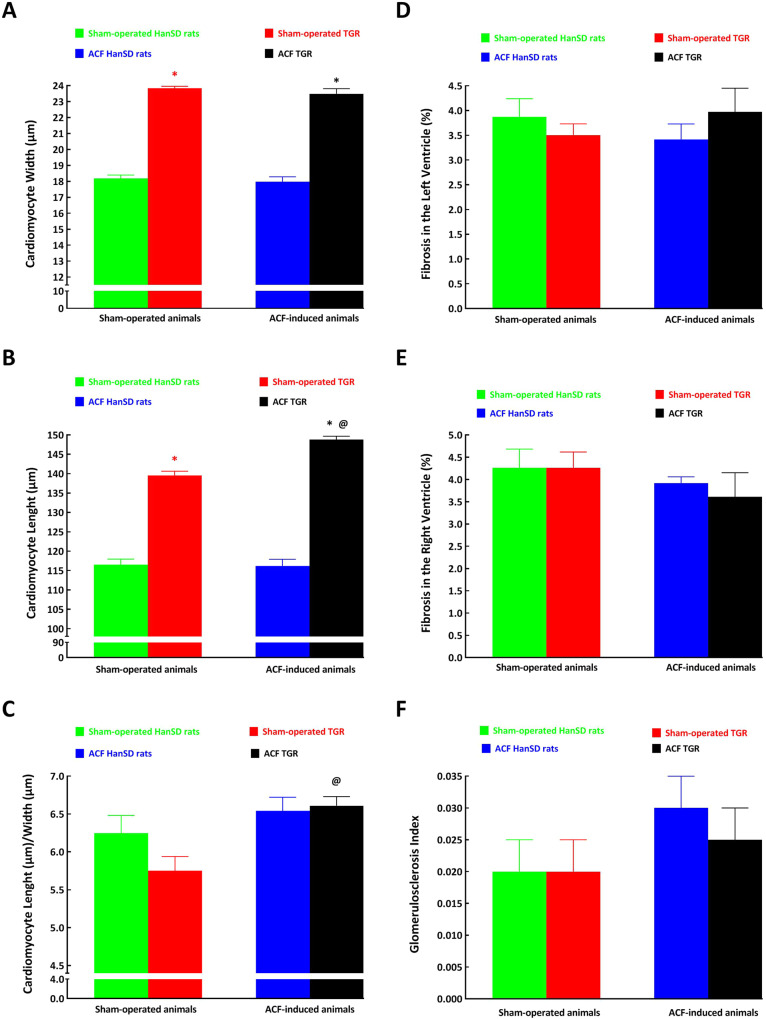
Cardiomyocyte width (**A**), cardiomyocyte length (**B**), ratio of cardiomyocyte length to cardiomyocyte width (**C**), fibrosis in the left ventricle (**D**), fibrosis in the right ventricle (**E**) and glomerulosclerosis index (**F**) in sham-operated Hannover Sprague-Dawley (HanSD) rats (green bars), sham-operated Ren-2 transgenic rats (TGR) (red bars), HanSD rats with aorto-caval fistula (ACF) (blue bars) an ACF TGR (black bars), as recorded one week after sham-operation or ACF creation. *p < 0.05 compared with the data for sham-operated counterparts. ^**@**^*p* < 0.05 compared with sham-operated TGR. Symbols are always shown in the color appropriate for the corresponding experimental group

Figure 2 summarizes the morphological changes at the cardiomyocyte level. Sham-operated TGR showed significantly higher cardiomyocyte width as compared with sham-operated HanSD rats (Fig. 2A), which was accompanied by higher cardiomyocyte length in sham-operated TGR than in sham-operated HanSD rats (Fig. 2B). This resulted in the similar ratio of cardiomyocyte length to cardiomyocyte width in sham-operated TGR and sham-operated HanSD rats (Fig. 2C). These findings at the cardiomyocyte level confirm that sham-operated TGR showed concentric LV hypertrophy. ACF creation did not change any of those parameters in HanSD rats. In contrast, ACF creation did not significantly change cardiomyocyte width in TGR, but caused a significant rise in cardiomyocyte length in TGR, which resulted in a significant increase of the ratio of cardiomyocyte length to cardiomyocyte width in ACF TGR as compared with sham-operated TGR (Fig. 2A–C). These findings at the cardiomyocyte level corroborate the observation from the whole organ level and suggest that ACF TGR are in the phase of development of extensive eccentric LV hypertrophy, which is a hallmark for this model of HF, already seen in this very early phase. Figure 2D, E summarize the data on the index of myocardial fibrosis (expressed in %) in the LV and RV. There were no significant differences in the myocardial fibrosis in the LV or the RV between sham-operated HanSD rats and sham-operated TGR and ACF induction did not alter the degree of myocardial fibrosis in ACF HanSD rats or ACF TGR. There were no signs of myocardial necrosis or appearance of scar tissue in any experimental group.

As shown in Fig. 2F, there were no significant differences in GSI between experimental groups and it is noteworthy that all values for GSI were low, clearly in the range which is regarded normal for healthy renal parenchyma. The same is valid for TSI (data not shown).

**Fig. 3:**
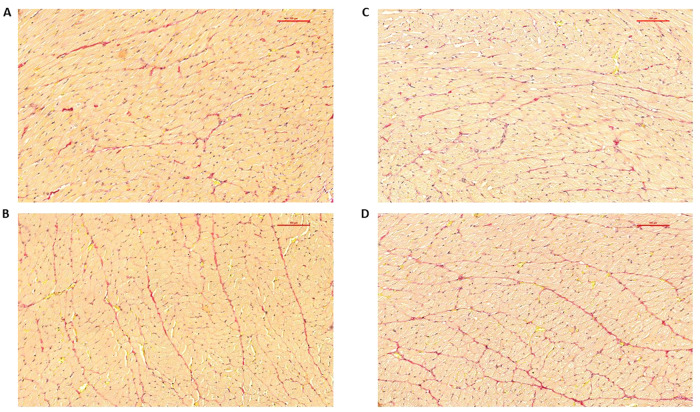
Representative histological images of the left ventricle from the sham-operated Hannover Sprague-Dawley (HanSD) rats (**A**), sham-operated Ren-2 transgenic rats (TGR) (**B**), HanSD rats with aorto-caval fistula (ACF) (**C**) and ACF TGR (**D**), recorded one week after sham-operation or ACF creation. Sections are stained with Picrosirius Red (200x), in these bright-field microscopy images, the collagen is red against a pale yellow background. The scale bar in the figure is 100 µm

**Fig. 4:**
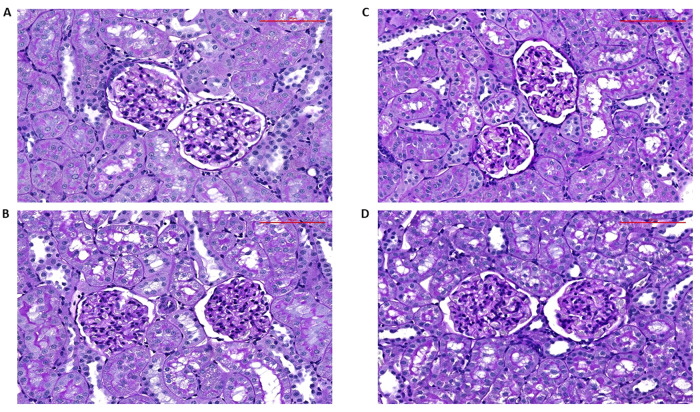
Representative histological images of the renal cortex from the sham-operated Hannover Sprague-Dawley (HanSD) rats (**A**), sham-operated Ren-2 transgenic rats (TGR) (**B**), HanSD rats with aorto-caval fistula (ACF) (**C**) and ACF TGR (**D**), recorded one week after sham-operation or ACF creation. Sections are stained with periodic acid, for Schiff reaction. The scale bar in the figure is 100 µm

Representative histological images of myocardial fibrosis in the LV are shown in Fig. 3, and representative images of the renal parenchyma are shown in Fig. 4.

**Fig. 5:**
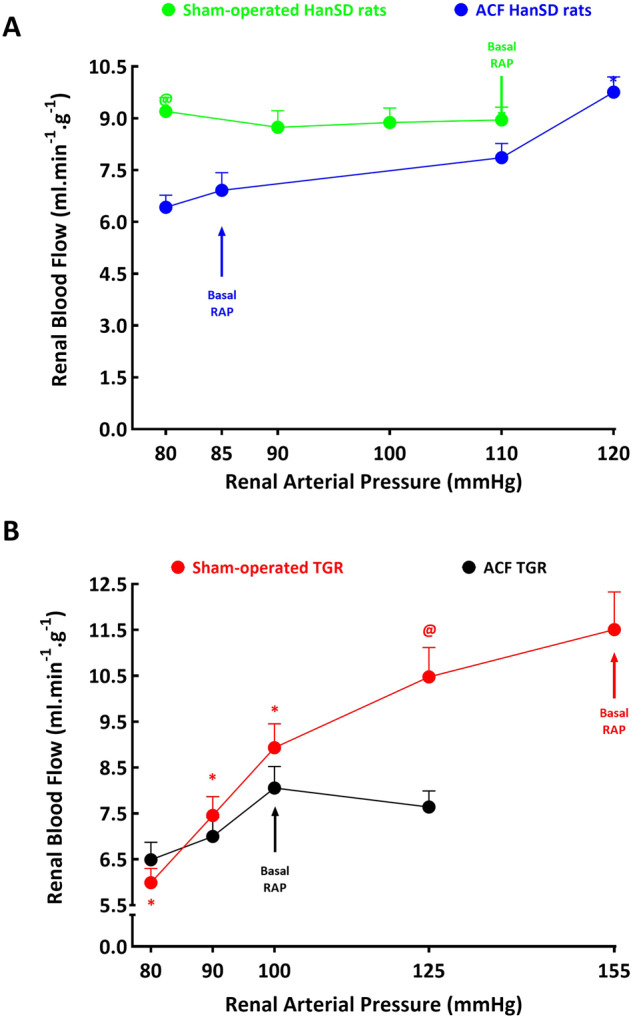
Relationship between renal arterial pressure (RAP) and renal blood flow in (**A**) sham-operated Hannover Sprague-Dawley (HanSD) rats (green line) and HanSD rats with aorto-caval fistula (ACF) (blue line), (**B**) in sham-operated Ren-2 transgenic rats (TGR) (red line) and ACF TGR (black line) in animals exposed to experimental protocol. *p < 0.05 compared with the values at the basal RAP, ^**@**^*p* < 0.05 compared with corresponding values either at the same RAP (for experimental protocol) or at the same time point (for control protocol). Symbols are always shown in the color appropriate for the corresponding experimental group

The relation of RBF to actual RAP is shown in Fig. 5. It is seen that sham-operated HanSD rats as well as ACF HanSD rats maintained autoregulatory capacity of RBF in response to changes of RAP even in case of its lowest level (80 mmHg) (Fig. 5A). Only the increase of RAP in ACF HanSD rats to 120 mmHg significantly increased RBF. RBF tended to decrease with RAP in ACF HanSD rats, but the changes did not reach significance level (*p* = 0.058).

As shown in Fig. 5B, sham-operated TGR exhibited impaired autoregulatory capacity of RBF, because reduction of RAP to 100 mmHg was sufficient to elicit significant blood flow decreases when compared with the basal values of RAP; these changes were much more pronounced at 80 mmHg. In contrast, in ACF TGR similar changes in RAP, i.e. RAP reductions to 90 and 80 mmHg or an increase to 125 mmHg only tended to affect alterations in RBF (*p* = 0.061, *p* = 0.059 and *p* = 0.062 changes not significant in all cases).

In all experimental groups that were exposed to the time control protocol (five 30-min periods without alterations of RAP), the rats showed marked and similar volume expansion (haematocrit values decreased from 49 ± 4% to 36 ± 3%; *p* < 0.05). Only in ACF HanSD rats did RBF increase significantly in the final period of the control study, to values that were almost identical with those in sham-operated HanSD rats. Otherwise no significant changes in RBF were seen in the animals exposed to time control protocol (data not shown). Nor did the course of MAP significantly change in the animals subjected to the control protocol (again, data not shown).

**Fig. 6:**
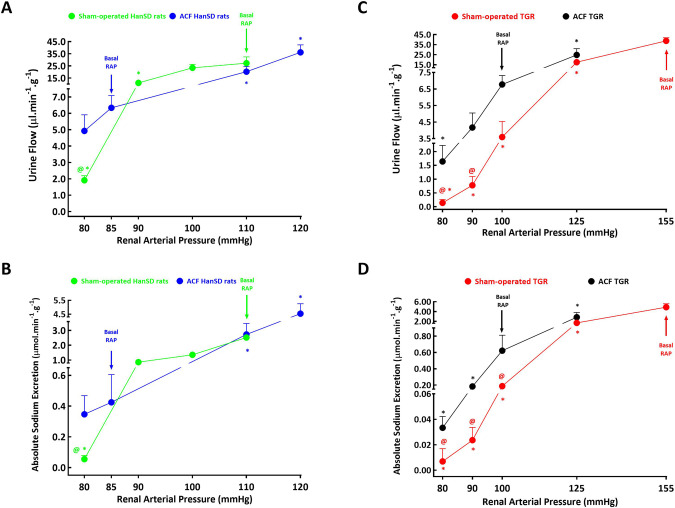
Relationship between renal arterial pressure and urine flow (**A** and **C**) and absolute sodium excretion (**B** and **D**) in sham-operated Hannover Sprague-Dawley (HanSD) rats (green line) and HanSD rats with aorto-caval fistula (ACF) (blue line), in sham-operated Ren-2 transgenic rats (TGR) (red line) and ACF TGR (black line) in animals exposed to experimental protocol. *p < 0.05 compared with the values at the basal RAP, ^**@**^*p* < 0.05 compared with the corresponding values at the same RAP. Symbols are always shown in the color appropriate for the corresponding experimental group. NB. Please notice and take into consideration the interrupted *y*-axes and the parameter curves

The relation of the parameters of renal excretion to RAP is shown in Fig. 6. The stepwise reductions in RAP in sham-operated HanSD rats caused significant decreases in urine flow and in absolute sodium excretion. However, in ACF HanSD rats the reduction in RAP from the basal 85 mmHg to the lowest level (80 mmHg) did not cause significant decreases in the excretory parameters; after RAP reduction all the values were significantly higher than in sham-operated HanSD rats at this RAP level. The stepwise increases in RAP in ACF HanSD rats, to 110 and 120 mmHg, elicited marked increases in urine flow and absolute sodium excretion. Consequently, at 110 mmHg (the basal level for sham-operated HanSD rats), there were no significant differences between ACF HanSD and sham-operated rats, even though at the basal level of RAP the values in ACF HanSD rats were markedly lower (Fig. 6A, B).

As shown in Fig. 6C, D, in sham-operated TGR as well as in ACF TGR stepwise reductions in RAP resulted in marked decreases in urine flow and absolute sodium excretion, however, in the latter rats the decreases from 90 to 80 mmHg were significantly less pronounced. Moreover, at the RAP of 100 of mmHg, the usual level for ACF TGR, the values of urine flow and absolute sodium excretion were substantially higher than the values measured when RAP was reduced to this level in sham-operated TGR. Moreover, when in ACF TGR their RAP was increased to 125 mmHg, the excretory parameters appeared higher (*p* = 0.058, not significantly different) than observed in sham-operated TGR at the same RAP level.

In all experimental groups undergoing time control protocol, urine flow and particularly absolute sodium excretion increased throughout the experiment (data not shown). However, when the changes were analyzed in percentage terms, no significant inter-group differences were observed.

## Discussion

The most important findings of our present study are as follows: (i) One week after induction of ACF, ACF TGR were in the process of development of eccentric LV hypertrophy whereas ACF HanSD rats still exhibited signs of LV concentric hypertrophy, (ii) One week after ACF creation ACF HanSD displayed bilateral cardiac hypertrophy whereas the increase in the whole HW in ACF TGR was exclusively due to RV hypertrophy, (iii) One week after ACF creation, ACF HanSD rats as well as ACF TGR showed lower MAP, RBF, urine flow and absolute sodium excretion as compared with their sham-operated (no ACF) counterparts, (iv) One week after ACF induction, ACF HanSD rats as well as ACF TGR demonstrated well-maintained RBF autoregulatory capacity and improved slope of the pressure-natriuresis relationship as compared with their sham-operated counterparts; the difference was more pronounced in ACF TGR than in ACF HanSD rats.

These findings require extensive discussion.

The first important set of findings presents analysis of the organ weights, blood pressure and renal function data. Earlier studies that have correlated the organ morphometry (HW, RVW and LVW) with cardiac function (evaluated by echocardiography and direct hemodynamic pressure-volume analyses) have shown that organ weights are reliable predictors for the onset of cardiac decompensation, at least in the high-output HF model obtained by ACF creation [25, 48–52]. In TGR the earliest phase of ACF-induced HF starts one week after ACF creation. Our recent studies [22, 54, 55] showed that in ACF TGR the onset of the decompensation phase occurs three to four weeks after ACF creation, as indicated by a clear onset of mortality. We showed that in HanSD rats, the decompensation phase of HF starts twenty weeks after creation of ACF [29, 30, 56]. Thus, the data obtained one week after ACF creation are certainly within compensation period, perhaps the earliest phase of HF. They indicate that as soon as one week after ACF creation, HanSD rats as well as TGR show marked increases in HW, however, the difference between HanSD rats and TGR is here noteworthy.

Our analysis at the whole organ level shows that ACF HanSD rats displayed bilateral cardiac hypertrophy, even though RV hypertrophy was here more pronounced (see Table 2). In contrast, in ACF TGR the increase in whole HW was exclusively due to an increase in RV mass (+78% as compared with sham-operated counterparts), whereas the LV mass increase was only about 2% of that in sham-operated TGR. The finding that RV hypertrophy in ACF HanSD rats and particularly in ACF TGR is markedly higher than is LV hypertrophy is of special interest. In our recent study creation of ACF resulted (within 3 weeks i.e. at the onset of the decompensation phase of HF in ACF TGR) in marked elevation in RV systolic pressure (RVSP) in ACF TGR (a surrogate for the RV afterload) [22]. Our observation is in agreement with a much earlier evidence that in normotensive rats creation of ACF resulted (within 4 weeks) in more pronounced RV hypertrophy [48] which was accompanied by elevation in RVSP. Closing the ACF resulted in normalization of LV as well as RV weights and RVSP, returning them to values observed in sham-operated normotensive rats. Therefore, it is believed that the relatively higher mass of RV compared with LV in ACF model of HF is the consequence of combination of volume overload and increased RV afterload. Even though it is tempting to claim that the higher degree of RV hypertrophy that we observed in TGR after ACF creation could be related to the level of RVSP higher than observed in ACF HanSD rats, our recent data do not support this notion. Actually, we found that the degree RVSP in ACF TGR (3 weeks after ACF creation) [22] and in normotensive rats (4 weeks after ACF creation) [42] was virtually identical. Therefore, it is doubtful that the higher degree of RV hypertrophy in ACF TGR than observed in ACF HanSD rats could be simply ascribed to higher RV afterload within the first week. Nevertheless, to conclusively resolve this issue more comprehensive studies are needed.

Our analysis at the tissue level showed that the heart remodeling response to chronic volume overload, i.e. development of eccentric LV hypertrophy (hallmark of the pathophysiology of HF in this model [49–53]) was clearly present in ACF TGR already one week after creation of ACF. Of special interest was that increased cardiomyocyte length in ACF TGR was here already present at this very early phase, whereas it was absent in ACF HanSD rats. We and others [57, 58] demonstrated that in normotensive rats exposed to chronic volume overload via ACF creation did not increase the cardiomyocyte length during first 60 days of volume overload. This indicates that in contrast to ACF HanSD rats ACF TGR were already in the process of development of extensive eccentric LV hypertrophy, whereas ACF HanSD rats still exhibited signs of LV concentric hypertrophy. Whether this difference in the LV hypertrophy pattern after ACF creation contributing to the different course of HF in ACF TGR and ACF HanSD rats remains uncertain and future studies are needed to address this issue. Moreover, our present findings show that one week after creation of ACF neither ACF HanSD rats nor ACF TGR showed increased myocardial fibrosis, in accordance with our previous studies in normotensive rats showing that even long-term chronic volume overload induced by ACF (11 and 21 weeks) did not cause significant rise in myocardial fibrosis [57], which strongly suggests that increased myocardial fibrosis does not play any important pathophysiological role in this model of HF.

Another important difference between ACF HanSD rats and ACF TGR is that the latter showed markedly greater lung congestion, which suggests that in TGR LV failure was developing very early after creation of ACF. It is not clear if the onset of LV failure was here related to the absence of significant LV hypertrophic response. According to the hypothesis first put forward by Linzbach [59], the transition to HF is triggered by a marked ventricular dilatation once the myocardial hypertrophic compensatory response is present. The Linzbach´s theory was confirmed in normotensive rats by Brower and Janicki [50] who showed that in the ACF-induced model of HF, RV hypertrophy has continued in response to volume overload: in contrast, the LV hypertrophic response reached the plateau eight weeks after ACF induction and then LV function began to deteriorate. Therefore, the authors concluded that the inability of the LV to further hypertrophy was one of the critical factors responsible for the development of overt HF in this model [50]. Nevertheless, a straightforward correlation between the degree of LV hypertrophy and development of overt HF is doubtful. Further studies evaluating cardiac morphometry and function by echocardiography and by pressure-volume analyses are needed to unequivocally confirm or refute the hypothesis that the absence of significant LV hypertrophic response is responsible for the marked lung congestion in ACF TGR in the very early phase after ACF creation.

In addition, our data show that already in the earliest HF stage both ACF TGR and ACF HanSD rats displayed substantially lowered RBF and renal excretory function, the latter seen from the low urine flow and absolute sodium excretion; evidently, renal dysfunction had been already established. These findings are important in that they further undermine the hypothesis that renal dysfunction is a simple consequence of hemodynamic disorder and/or excessive compensatory activation of the neurohormonal systems, such as the RAS and the sympathetic nervous system (SNS). More probably, the dysfunction is an important factor initiating the progression of HF. While the heart-kidney interactions in HF patients have been recognized for decades and reduced RBF and renal dysfunction have been shown to be important predictor of mortality in HF patients [10, 11, 60, 61], only more recently was the opinion expressed that the treatment of renal dysfunction itself can improve the long-term prognosis of patients with HF [62]. This new concept is in agreement with our present findings and the notion that kidney dysfunction is not merely a “victim” of HF progression but also, if not quite a “culprit”, at least an important factor in the pathophysiology of the progression of HF.

The second important set of findings relates to the autoregulatory capacity of the RBF and the pressure-natriuresis relationship. Studies over past five decades have unequivocally demonstrated that impaired renal autoregulation plays a critical role in the progression of various diseases, such as hypertension, chronic kidney disease and diabetes mellitus [63]. On the contrary, the disease models with maintained efficient renal autoregulation, as is the case with the spontaneously hypertensive rat (SHR), exhibit good protection against hypertension-induced renal injury [63]. In addition, numerous studies have shown that in all experimental animal models of hypertension, the pressure-natriuresis relationship is blunted compared with normotensive control animals [64–66]; this was specifically confirmed in several ANG II-dependent models of hypertension [37, 38, 40–42, 67, 68]. Even though ANG II is not a mediator of pressure-natriuresis, it is a powerful modulator of the slope of the pressure-natriuresis curve. Activation of the RAS can markedly suppress the pressure-natriuresis relationship, leading to an impairment of sodium excretion and progressive aggravation of hypertension, as indeed observed in ANG II-dependent models of hypertension [10, 69]. In this context, we found that in the Cyp1a1-Ren-2 transgenic rat [38], a model of inducible ANG II-dependent form of hypertension, an impairment of the autoregulation of renal hemodynamics and of the pressure-natriuresis relationship precedes the development of hypertension [38, 71–73], in agreement with the Guyton’s theory regarding the pathophysiology of the development of hypertension [74]. In view of such evidence and the findings reported mainly by Brenner´s and Frohlich´s groups [16–20] we put forward the hypothesis that impairment of autoregulation of the RBF and of the pressure-natriuresis relationship precedes the onset of the decompensation phase in high-output HF, and that this impairment is augmented in ANG II-dependent hypertension.

In agreement with previous studies, including our own [41, 75, 76], we found that sham-operated TGR displayed impairment of the RBF autoregulatory capacity and a marked suppression of the pressure-natriuresis relationship as compared with sham-operated HanSD rats. This would accord with the concept that such impairment is a critical mechanism in the pathophysiology of hypertension, as proposed by Guyton et al. [74] and supported by several other groups [36, 37, 63–68, 77]. These findings have supported our initial hypothesis about the role of the impairment of the renal autoregulation capacity and the pressure-natriuresis relationship in the onset of decompensation phase of HF, particularly in ACF TGR. It is bewildering, however, that ACF HanSD rats and ACF TGR did not exhibit impairment of RBF autoregulation and pressure-natriuresis relationship. ACF animals actually showed leftward shift of the pressure-natriuresis curve, especially prominent in ACF TGR. Thus, our data show that at the basal RAP level usual for ACF animals, marked reduction in RBF, urine flow, absolute sodium excretion was seen, as compared with the sham-operated (without ACF) counterparts. Although the well-maintained autoregulatory capacity of the RBF and the improved slope of the pressure-natriuresis relationship is incompatible with our original hypothesis, the data suggest that the leftward shift of the pressure-natriuresis curve might facilitate sodium excretion after ACF creation and help attenuate the reduction in sodium excretion under conditions of lower RAP in ACF animals, particularly in ACF TGR. In other words, if ACF TGR did not exhibit the leftward shift of the pressure-natriuresis relationship, a more pronounced impairment of sodium excretion would result, likely leading to faster progression of HF and the development of its overt form or even decompensation.

## Limitations of the study

The first limitation is the lack of data on plasma and kidney ANG II and atrial natriuretic peptide (ANP) levels. Activation of the RAS and ANP can substantially modulate the pressure-natriuresis relationship and RBF autoregulatory capacity [69, 70, 75–77], hence the value of knowing ANG II and ANP concentrations to estimate their role in the regulation of sodium excretion in the earliest phase of the high-output variant of HF. Unfortunately, it is impossible to obtain reliable data from such analyses in surgically stressed, anesthetized animals, as those required for the needed renal function studies. Plasma and tissue ANG II concentrations as well as renin secretion are in such animals higher than those measured in conscious rats after decapitation [32, 33, 78, 79]. Moreover, blood sampling itself for hormone determination would alter both BP and hormone activity and, obviously, obtaining reliable intrarenal hormone concentrations at various RAP levels is not feasible. Briefly, attempts to determine the hormone levels in our experimental setup would not be successful and this was not done in earlier studies [36–42, 64–68, 75–77].

Another limitation was the absence of assessment of the effects of pharmacological blockade of the RAS. Increased RAS activity is a co-determinant of the pathophysiology of progression in ACF-induced HF model [19–23, 25–28] and modulates the pressure-natriuresis relationship [69, 70]. Therefore it would be interesting to assess the effects of the blockade, especially in ACF TGR. Indeed, we found recently that 15-weeks’ blockade of the RAS with angiotensin-converting enzyme inhibitor (ACEi) attenuated eccentric cardiac hypertrophy and improved ejection fraction without restoring RBF [23], whereas the blockade of RAS by ANG II type 1 (AT_1_) receptor antagonist improved cardiac function and restored RBF. This implies beneficial effects of RAS blockade on the survival of hypertensive TGR with ACF-induced HF [25, 44, 45, 80] and indicates different effects of the blockade depending on the use of ACEi or AT_1_ receptor inhibition. Future studies are needed to address this issue, however, they would be difficult to perform: either blockade would decrease BP and hypotension is one of major adverse effects of pharmacological treatment in HF [3–6].

Nonetheless, even considering the aforementioned limitations, we are convinced that the present results provide important information on the pathophysiology of experimental high-output HF which is a reasonable model of human cardiorenal syndrome.

## Summary and conclusion

The present results show that even in the very early stage of high-output HF, renal dysfunction is demonstrable in originally normotensive as well as in hypertensive rats. However, the dysfunction and the subsequent HF decompensation cannot be simply ascribed to impairment of the autoregulatory capacity of the RBF and of the pressure-natriuresis relationship. On the contrary, in the high-output variant of HF in originally RAS-dependent hypertensive rats, RBF autoregulation is well-maintained and the slope of the pressure-natriuresis relationship is improved. This suggests that a compensatory mechanism is provided which attenuates impairment of renal sodium excretion. These findings should be considered in attempts to develop new treatment strategies in HF individuals, especially those displaying hypertension before the onset of HF.

## References

[1] Roger VL (2021). Epidemiology of heart failure. A contemporary perspective. Circ Res.

[2] Bullock H, Yellon DM, Hausenloy DJ (2016). Reducing myocardial infarct size: challenges and future opportunities. Heart.

[3] Mullens W, Verbrugge FH, Nijst P, Tang WHW (2017). Renal sodium avidity in heart failure: from pathophysiology to treatment strategies. Eur Heart J.

[4] Kassi M, Hannawi B, Trachtenberg B (2018). Recent advances in heart failure. Curr Opin Cardiol.

[5] Rangawwami J, Bhalla V, Blair JEA, Chang TI, Costa S, Lentine KL (2019). American Heart Asssociation Council on the Kidney in Cardiovascular Disease and Council on Clinical Cardiology. Cardiorenal syndrome: classification, pathophysiology, diagnosis, and treatment strategies. A scientific statement from the American Heart Association. Circulation.

[6] Mullens W, Damman K, Testani JM, Martens P, Mueller C, Lassus J (2020). Evaluation of kidney function throughout the heart failure trajectory – a position statement from the Heart Failure Association of the European Society of Cardiology. Eur J Heart Fail.

[7] Khayyat-Kholghi M, Oparil S, Davis BR, Tereshchenko LG (2021). Worsening kidney function is the major mechanism of heart failure in hypertension. The ALLHAT study. J Am Coll Cardiol HF.

[8] Hillege HL, Nitsch D, Pfeffer MA, Swedberg K, McMurray JJV, Yusuf S (2006). Renal function as a predictor of outcome in broad spectrum of patients with heart failure. Circulation.

[9] Packer M, Lee WH, Kessler PD (1986). Preservation of glomerular filtration rate in human heart failure by activation of the renin-angiotensin system. Circulation.

[10] Hillege HL, Girbes AR, de Kam PJ, Boomsma F, de Zeeuw D, Charlesworth A (2000). Renal function, neurohormonal activation, and survival in patients with chronic heart failure. Circulation.

[11] Jose P, Skali H, Anavekar N, Tomson C, Krumholz HM, Rouleau JL (2006). Increase in creatinine and cardiovascular risk in patients with systolic dysfunction after myocardial infarction. J Am Soc Nephrol.

[12] Packer M (1992). The neurohormonal hypothesis: a theory to explain the mechanism of disease progression in heart failure. J Am Coll Cardiol.

[13] Mann DL, Felker M (2021). Mechanisms and models in heart failure. A translation approach. Circ Res.

[14] Vallon V, Verma S (2021). Effects of SGLT2 inhibitors on kidney and cardiovascular function. Annu Rev Physiol.

[15] Barger AC, Muldowney FP, Liebowitz MR (1959). Role of the kidney in the pathogenesis of congestive heart failure. Circulation.

[16] Hostetter TH, Pfeffer JM, Pfeffer MA, Dworkin LD, Braunwald E, Brenner BM (1983). Cardiorenal hemodynamics and sodium excretion in rats with myocardial infarction. Am J Physiol.

[17] Ichikawa I, Pfeffer JM, Pfeffer MA, Hostetter TH, Brenner BM (1984). Role of angiotensin II in the altered renal function of congestive heart failure. Circ Res.

[18] Stanton RC, Brenner BM (1986). Role of kidney in congestive heart failure. Acta Med Scand.

[19] Numabe A, Hishikimi T, Komatsu K, Frohlich ED (1994). Intrarenal hemodynamics in low- and high-output cardiac failure in rats. Am J Med Sci.

[20] Nishikimi T, Frohlich ED (1993). Glomerular hemodynamics in aortocaval fistula rats: role of renin-angiotensin system. Am J Physiol.

[21] Vacková Š, Kikerlová S, Melenovský V, Kolář F, Imig JD, Kompanovska-Jezierska E (2019). Altered renal vascular responsiveness in rats with angiotensin II-dependent hypertension and congestive heart failure. Kidney Blood Press Res.

[22] Honetschlagerová Z, Škaroupková P, Kikerlová S, Vaňourková Z, Husková Z, Melenovský V (2021). Renal sympathetic denervation attenuates congestive heart failure in angiotensin II-dependent hypertension: studies with Ren-2 transgenic hypertensive rats with aorto-caval fistula. Kidney Blood Press Res.

[23] Kratky V, Vanourkova Z, Sykora M, Szeiffova Bacova B, Hruskova Z, Kikerlova S (2021). AT_1_ receptor blocker, but not an ACE inhibitor, prevents kidneys from hypoperfusion during congestive heart failure in normotensive and hypertensive rats. Sci Rep.

[24] Kilkenny C, Browne WJ, Cuthill IC, Emerson M, Altman DC (2010). Improving bioscience research reporting: the ARRIVE guidelines for reporting animal research. J Pharm Pharmacother.

[25] Červenka L, Melenovský V, Husková Z, Škaroupková P, Nishiyama A, Sadowski J (2015). Inhibition of soluble epoxide hydrolase counteracts the development of renal dysfunction and progression of congestive heart failure in Ren-2 transgenic hypertensive rats with aorto-caval fistula. Clin Exp Pharm Physiol.

[26] Honetschlagerová Z, Škaroupková P, Kikerlová S, Husková Z, Maxová H, Melenovský V (2021). Effects of renal sympathetic denervation on the course of congestive heart failure combined with chronic kidney disease: insight from studies with fawn-hooded hypertensive rats with volume overload induced using aorto-caval fistula. Clin Exp Hypertens.

[27] Winaver J, Hoffman A, Burnett JC, Haramati A (1988). Hormonal determinants of sodium excretion in rats with experimental high-output heart failure. Am J Physiol.

[28] Abassi Z, Goltsman I, Karram T, Winaver J, Hoffman A (2011). Aortocaval fistula in rat: a unique model of volume-overload congestive heart failure and cardiac hypertrophy. J Biomed Biotechnol.

[29] Kratky V, Kopkan L, Kikerlova S, Huskova Z, Taborsky M, Sadowski J (2018). The role of renal vascular reactivity in the development of renal dysfunction in compensated and decompensated congestive heart failure. Kidney Blood Press Res.

[30] Melenovsky V, Skaroupkova P, Benes J, Torresova V, Kopkan L, Cervenka L (2012). The course of heart failure development and mortality in rats with volume overload due to aorto-caval fistula. Kidney Blood Press Res.

[31] Červenka L, Wang C-T, Navar LG (1998). Effects of acute AT1 receptor blockade by candesartan on arterial pressure and renal function in rats. Am J Physiol.

[32] Husková Z, Kramer HJ, Vaňourková Z, Červenka L (2006). Effects of changes in sodium balance on plasma and kidney angiotensin II levels in anesthetized and conscious Ren-2 transgenic rats. J Hypertens.

[33] Kopkan L, Kramer HJ, Huskova Z, Vaňourková Z, Škaroupková P, Thumová M (2005). The role of intrarenal angiotensin II in the development of hypertension in Ren-2 transgenic rats. J Hypertens.

[34] Kopkan L, Husková Z, Vanourková Z, Thumová M, Skaroupková P, Cervenka L (2007). Superoxide and its interaction with nitric oxide modulates renal function in prehypertensive Ren-2 transgenic rats. J Hypertens.

[35] Jíchová Š, Kopkan L, Husková Z, Doleželová Š, Neckář J, Kujal P (2016). Epoxyeicosatrienoic acid analog attenuates the development of malignant hypertension, but does not reverse it once established: a study in Cyp1a1-Ren-2 transgenic rats. J Hypertens.

[36] Roman RJ, Cowley AW (1985). Characterization of a new model for the study of pressure-natriuresis in the rat. Am J Physiol.

[37] Wang CT, Chin SY, Navar LG (2000). Impairment of pressure-natriuresis and renal autoregulation in ANG II-infused hypertensive rats. Am J Physiol.

[38] Erbanová M, Thumová M, Husková Z, Vaněčková I, Vaňourková Z, Mullins JJ (2009). Impairment of the autoregulation of renal hemodynamics and of the pressure-natriuresis relationship precedes the development of hypertension in Cyp1a1-Ren-2 transgenic rats. J Hypertens.

[39] Sporková A, Kopkan L, Vacarbová Š, Husková Z, Hwang SH, Hammock BD (2011). Role of cytochrome P-450 metabolites in the regulation of renal function and blood pressure 2-kidney, 1-clip hypertensive rats. Am J Physiol.

[40] Honetschlagerová Z, Sporková A, Kopkan L, Husková Z, Hwang SH, Hammock BD (2011). Inhibition of soluble epoxide hydrolase improves the impaired pressure-natriuresis relationship and attenuates the development of hypertension and hypertension-associated end-organ damage in Cyp1a1-Ren-2 transgenic rats. J Hypertens.

[41] Varcabová Š, Husková Z, Kramer HJ, Hwang HS, Hammock BD, Imig JD (2013). Antihypertensive action of soluble epoxide hydrolase inhibition in Ren-2 transgenic rats is mediated by suppression of the intrarenal renin-angiotensin system. Clin Exp Pharm Physiol.

[42] Honetschlagerová Z, Kitada K, Husková Z, Sporková A, Kopkan L, Bürgelová M (2013). Antihypertensive and renoprotective actions of soluble epoxide hydrolase inhibition in ANG II-dependent malignant hypertension are abolished by pretreatment with L-NAME. J Hypertens.

[43] Pokorný M, Mrázová I, Šochman J, Melenovský V, Malý J, Pirk J (2018). Isovolumic loading of the failing heart by intraventricular placement of a spring expander attenuates cardiac atrophy after heterotopic heart transplantation. Biosci Rep.

[44] Kala P, Vaňourková Z, Škaroupková P, Kompanowska-Jezierska E, Sadowski J, Walkowska A (2023). Endothelin type A receptor blockade increases renoprotection in congestive heart failure combined with chronic kidney disease: studies in 5/6 nephrectomized rats with aorto-caval fistula. Biomed Pharmacother.

[45] Gawrys O, Husková Z, Škaroupková P, Honetschlägerová Z, Vaňourková Z, Kikerlová S, et al. The treatment with sGC stimulator improves survival of hypertensive rats in response to volume-overload induced by aorto-caval fistula [published online ahead of print, 2023 Jun 20]. Naunyn Schmiedebergs Arch Pharmacol. 2023; 10.1007/s00210-023-02561-y. 10.1007/s00210-023-02561-y PMC10643302 37338578

[46] Obayashi M, Yano M, Kohno M, Kobayashi S, Tanigawa T, Hironaka K (1997). Dose-dependent effect of ANG II-receptor antagonist on myocyte remodeling in rat cardiac hypertrophy. Am J Physiol.

[47] Nakano Y, Hirano T, Uehara K, Nishibayashi S, Hattori K, Aihara M (2008). New rat model induced by anti-glomerular basement membrane antibody shows severe glomerular adhesion in early stage and quickly progresses to end-stage renal failure. Pathol Int.

[48] Gerdes AM, Clark LC, Capassso JM (1995). Regression of cardiac hypertrophy after closing and aorto-caval fistula in rats. Am J Physiol.

[49] Brower GL, Henegar JR, Janicki JS (1996). Temporal evaluation of left ventricular remodeling and function in rats with chronic volume overload. Am J Physiol.

[50] Brower GL, Janicki JS (2001). Contribution of ventricular remodeling to pathogenesis of heart failure in rats. Am J Physiol.

[51] Wang X, Ren B, Liu S, Sentex E, Tappia PS, Dhalla NS (2003). Characterization of cardiac hypertrophy and heart failure due to volume overload in the rat. J Appl Physiol.

[52] Oliver-Dussault C, Ascah A, Marcil M, Matas J, Picard S, Pibarot B (2010). Early predictors of cardiac decompensation in experimental volume overload. Mol Cell Biochem.

[53] Hutchinson KR, Guggilam A, Cismowski MJ, Galantowics ML, West TA, Stewart JA (2011). Temporal pattern of left ventricle structural and functional remodeling following reversal of volume overload heart failure. J Appl Physiol.

[54] Kala P, Miklovič M, Jíchová Š, Škaroupková P, Vaňourková Z, Maxová H (2021). Effects of Epoxyeicosatrienoic acid-enhancing therapy on the course of congestive heart failure in angiotensin II-dependent rat hypertension: from mRNA analysis towards functional in vivo evaluation. Biomedicines.

[55] Kala P, Červenka L, Škaroupková P, Táborský M, Kompanowska-Jezierska E, Sadowski J (2019). Sex-linked differences in the mortality in Ren-2 transgenic hypertensive rats with aorto-caval fistula: effects of treatment with angiotensin converting enzyme alone and combined with inhibitor of soluble epoxide hydrolase. Physiol Res.

[56] Krátký V, Kikerlová S, Husková Z, Sadowski J, Kolář F, Červenka L (2019). Enhanced renal vascular responsiveness to angiotensin II and norepinephrine: a unique feature of female rats with congestive heart failure. Kidney Blood Press Res.

[57] Benes J, Melenovsky V, Skaroupkova P, Pospisilova J, Petrak J, Cervenka L (2011). Myocardial morphological characteristics and proarrhythmic substrate in the rat model of heart failure due to chronic volume overload. Anat Rec (Hoboken).

[58] Du Y, Plante E, Janicki JS, Brower GL (2010). Temporal evaluation of cardiac myocyte hypertrophy and hyperplasia in male rats secondary to chronic volume overload. Am J Pathol.

[59] Linzbach AJ (1960). Heart failure from the point of view of quantitative anatomy. Am J Cardiol.

[60] Goldberg A, Hammerman H, Petcherski S, Zdorovyak A, Yalonetsky S, Kapeliovich M (2005). Inhospital and 1-year mortality of patients who develop worsening renal function following acute ST-elevation myocardial infarction. Am Heart J.

[61] Rangawwami J, Bhalla V, Blair JEA, Chang TI, Costa S, Lentine KL (2019). American Heart Asssociation Council on the Kidney in cardiovascular disease and council on clinical cardiology. Circulation.

[62] Ciccarelli M, Dawson D, Facao-Pires I, Giacca M, Hamdani N, Heymans S (2021). Reciprocal organ interactions during heart failure: a position paper from the ESC Working Group on Myocardial Function. Cardiovasc Res.

[63] Carlstrom M, Wilcox CS, Arendshorst WJ (2015). Renal autoregulation in health and disease. Physiol Rev.

[64] Roman RJ, Cowley AW (1985). Abnormal pressure-diuresis-natriuresis response in spontaneously hypertensive rats. Am J Physiol.

[65] Roman RJ (1986). Abnormal renal hemodynamics and pressure-natriuresis relationship in Dahl salt-sensitive rats. Am J Physiol.

[66] Miao CY, Liu KL, Benzoni D, Sassard J (2005). Acute pressure-natriuresis function shows early impairment in Lyon hypertensive rats. J Hypertens.

[67] Ploth DW, Roy RN, Huang WC, Navar LG (1981). Impaired renal blood flow and cortical pressure autoregulation in contralateral kidneys of Goldblatt hypertensive rats. Hypertension.

[68] Van der Mark J, Kline RL (1994). Altered pressure natriuresis in chronic angiotensin II hypertension in rats. Am J Physiol.

[69] Mitchell KD, Navar LG. Intrarenal actions of angiotensin II in the pathogenesis of experimental hypertension. In: Laragh JH, Brenner BM, editors. Hypertension: pathophysiology, diagnosis and management. New York, NY, Raven Press, Publishers, 1990; pp. 1437–1452.

[70] Hall JE, Brans MV, Henegar JR (1999). Angiotensin II and long-term arterial pressure regulation: the overriding dominance of the kidney. J Am Soc Nephrol.

[71] Kantachuvesiri S, Fleming S, Peters J, Peters B, Brooker G, Lammie AG (2001). Controlled hypertension, a transgenic toggle switch reveals differential mechanisms underlying vascular disease. J Biol Chem.

[72] Vaňourková Z, Kramer HJ, Husková Z, Vaněčkováková I, Opočenský M, Čertíková Chábová V (2006). AT1 receptor blockade is superior to conventional triple therapy in protecting against end-organ damage Cyp1a1-Ren-2 transgenic rats with inducible hypertension. J Hypertens.

[73] Mitchell KD, Bagatell SJ, Miller CS, Mouton CR, Seth DM, Mullins JJ (2006). Genetic clamping of renin gene expression induces hypertension and elevation of intrarenal II levels of graded severity in Cyp1a1-Ren2 transgenic rats. JRAAS.

[74] Guyton AC, Hall JE, Coleman TG, Manning RD Jr. The dominant role of the kidneys in the long term regulation of arterial pressure in normal and hypertensive states. In: Laragh JH, Brenner BM, editors. Hypertension: pathophysiology, diagnosis and management. New York, NY, Raven Press, Publishers, 1990; pp. 1029–1052.

[75] Lippoldt A, Gross V, Bohlender J, Ganten U, Luft FC (1996). Lifelong angiotensin-converting enzyme inhibition, pressure natriuresis, and renin-angiotensin system gene expression in transgenic (mRen-2)27 rats. J Am Soc Nephrol.

[76] Springate J, Van Liew J, Ganten D (1997). Enalapril and pressure-diuresis in hypertensive rats transgenic for mouse renin gene. Kidney Blood Press Res.

[77] Hall JE, Mizelle HL, brands MV, Hildenbrandt DA (1992). Pressure natriuresis and angiotensin II in reduced kidney mass, salt-induced hypertension. Am J Physiol.

[78] Fox J, Guan S, Hymel AA, Navar LG (1992). Dietary Na and ACE inhibition effects on renal tissue angiotensin I and II and ACE activity in rats. Am J Physiol.

[79] Husková Z, Kramer HJ, Thumová M, Vanourková Z, Bürgelová M, Teplan V (2006). Effects of anesthesia on plasma and kidney ANG II levels in normotensive and ANG II-dependent hypertensive rats. Kidney Blood Press Res.

[80] Kala P, Gawrys O, Miklovič M, Vaňourková Z, Škaroupková P, Jíchová Š (2023). Endothelin type A receptor blockade attenuates aorto-caval fistula-induced heart failure in rats with angiotensin II-dependent hypertension. J Hypertens.

